# Quality characteristics of soybean fermented by *Mucor*, *Rhizopus*, and *Aspergillus* from *meju*

**DOI:** 10.1016/j.heliyon.2023.e14092

**Published:** 2023-02-27

**Authors:** Sojeong Heo, Junghyun Park, Kwang-Geun Lee, Jong-Hoon Lee, Do-Won Jeong

**Affiliations:** a Department of Food and Nutrition, Dongduk Women's University, Seoul, 02748, Republic of Korea; b Department of Food Science and Biotechnology, Dongguk University-Seoul, Goyang, 10326, Republic of Korea; c Department of Food Science and Biotechnology, Kyonggi University, Suwon, 16227, Republic of Korea

**Keywords:** Fermented soybean, *Aspergillus oryzae*, *Mucor* sp., *Rhizopus oryzae*, Fermentation

## Abstract

Three candidate starter strains—*Aspergillus oryzae* SNU-G, *Mucor* sp. KACC 46077, and *Rhizopus oryzae* KACC 40256—were inoculated into soybean, as individual strains or in combination, to assess their roles in fermentation. All the strains increased the pH, amino-type nitrogen, and moisture content of the soybean during fermentation, and decreased the lightness, redness, and yellowness. The inoculated strains increased to an average density of 1.37 × 10^8^ spores/g (from the initial 5.0 × 10^7^ spores/g) after 20 days of fermentation. Forty-two volatile compounds, including an acid, alcohols, carbonyls, furans, and a pyrazine, were more abundant in soybean fermented with starters than in controls. *A. oryzae* SNU-G increased the pH more than the other strains and produced more volatile alcohol compounds. *R. oryzae* KACC 40256 resulted in the lowest reduction of redness and yellowness during the fermentation and produced large amounts of carbonyl compounds, including two specific volatile compounds, 2-hydroxy-3-methylcyclopent-2-en-1-one and (3*E*)-3-ethyl-2-methylhexa-1,3-diene. *Mucor* sp. KACC 46077 contributed the least to pH change and volatile compound production, and did not produce specific volatile compounds. Although no significant synergy in the production of volatile compounds was found when using mixtures of strains compared with application of single strains, the quality of fermented soybeans was confirmed to be different depending on the strain(s) applied.

## 1 Introduction

*Meju*, a naturally fermented soybean product, is prepared by soaking, steaming, crushing, and molding soybean into blocks and allowing them to ripen for 1–2 months. The ripened *meju* is a complex microbial ecosystem; the microbes are responsible for macromolecular degradation in the *meju* via proteolysis, glycolysis, and lipolysis. *Meju* is a main ingredient of *doenjang*, which is produced by fermentation over 6 months of the mashed solid portion with high-salt (approximately 18%) brine [1,2].

*Doenjang* is a salty, sweet, earthy, fruity, and savory fermented soybean paste that has been used as a condiment in Korea for a long time. The unique flavor of *doenjang* comes from the taste of amino acids produced by the decomposition of soybean protein by microbial enzyme reactions during the fermentation and maturation processes [3,4]. Therefore, many studies have focused on the microbial community in the traditional fermentation of *doenjang* [[5], [6], [7]]. Several reports have focused on the sensory and metabolite changes during *doenjang* ripening [8,9]; however, correlations between specific metabolites and microorganisms are still incomplete. In addition, because of the complexities of multiple strains working together and the different microbial communities in spontaneous fermentations such as that of traditional *doenjang*, it has been difficult to organize and reproduce systems for industrialization with reliable quality.

Studies have assessed the effects of *meju* shapes and strains on the quality of traditional *doenjang* [10]; on *doenjang* preparation using koji (mold and bacteria cultivated with carbohydrate material such as rice, using *Aspergillus oryzae* or/and *Bacillus subtilis*) [11]; and on the improvement of the quality of traditional *doenjang* by using a mixture of yeasts, including *Saccharomyces rouxii* [12]. Control of *meju* fermentation is important for the quality of *doenjang*.

For the production of traditional *doenjang* with standardized and sanitary qualities, modern manufacturing processes use selected *Aspergillus* spp. that have high amylase and protease activities. Although *Mucor* spp. and *Rhizopus oryzae* were found to be more dominant than *Aspergillus* spp. in culture-independent analyses of the microbial communities of fermented soybean products [13,14], *A. oryzae* is usually used in commercial production using starter culture such as *koji*, a culture of *A. oryzae* on grains, and *Mucor* spp. and *Rhizopus oryzae* have not been considered as starters in manufacturing or research. Therefore, in this study, we analyzed the influence of each of *A. oryzae*, *Mucor* sp., and *R. oryzae*, as well as their combinations, on soybean fermentation in a model system.

## 2 Materials and methods

### 2.1 Strains and culture conditions

*A. oryzae* SNU-G, *Mucor* sp. KACC 46077, and *R. oryzae* KACC 40256 were used for soybean fermentation. *A. oryzae* SNU-G, isolated from industrial *doenjang* koji, was kindly provided by Prof. Lee (Kookmin University, Korea) [15]. *Mucor* sp. KACC 46077 and *R. oryzae* KACC 40256 from *meju* were obtained from the Korean Agricultural Culture Collection (KACC). The strains were cultured on potato–dextrose–agar (PDA; Becton, Dickinson and company, USA) for 3 days at 30 °C. Spores were harvested using 0.1% Tween 80 buffer after culturing the strains on PDA plates.

### 2.2 Inoculation of fungi in model soybean fermentation

Previously, we established a model system for production of commercial *doenjang* using starter cultures [16]. Since salt was not added in the model system, a commercial fermented soybean system in the same state as the salt concentration of *meju* was applied to the experiment. Soybean (250 g) was washed and soaked in the same volume of water for 12 h and autoclaved for 30 min at 121 °C in a 1-L reagent bottle. The soybean was then cooled to approximately 30 °C. Cells cultured on PDA were inoculated into the sterilized soybean at 5 × 10^7^ spores/g and mixed thoroughly. In mixed strains, a total of 5 × 10^7^ spores/g divided equally between the two or three strains were inoculated. The inoculated soybean samples were incubated aseptically at 25 °C for 20 days. Sterilized soybean was used as a control and incubated in the same conditions. Sampling of 50 g was performed every 5 days and samples were stored at −80 °C until volatile compound analysis. To verify reproducibility, each fermented sample was produced in duplicate at the same time.

### 2.3 Physicochemical constituent analysis

For pH measurement, 10-g samples were mixed slightly with 30 mL of deionized water for 5 min, and then the pH of the supernatant was measured using a pH meter (Thermo Fisher Scientific, USA). One gram of ground samples was analyzed using a colorimeter (Color JC-801S, Color Techno System Corporation, Japan); color was expressed in terms of the values lightness (L), redness (a), and yellowness (b). The NaCl content was measured by titration with silver nitrate according to the Mohr method [17]. Moisture content was measured using a drying oven (LDO-150F, Daihan LabTech Co., Ltd., Korea).

The analysis of amino-type nitrogen was performed using the formalin titration method [18]. Five grams of soybean were homogenized with 95 mL of distilled water and then centrifuged at 10,000×*g* for 5 min. Supernatants were adjusted to pH 8.4 by adding 0.1 M NaOH. Subsequently, neutral formalin (10 mL) and distilled water (10 mL) were added to the solution (10 mL). The mixture was titrated with 0.1 M NaOH to reach pH 8.4. The final titrated volume was used to calculate the amino-type nitrogen content. Distilled water was used as the test blank.

### 2.4 Growth rates of the inoculated starter candidates in soybean

Ten-gram samples were homogenized with 40 mL of sterilized peptone water and filtered through sterilized gauze. The filtrate was centrifuged at 1000×*g* for 5 min to precipitate insoluble material, and then the supernatant was diluted decimally in sterilized 0.1% (v/v) peptone water, spread on PDA, incubated at 30 °C for 5 days, and the spores were counted.

### 2.5 Evaluation of volatile compound production

Aroma compounds were absorbed into a silicone/Teflon septum (Supelco, USA) after a fermented sample (1 g) was mixed with 3.25 mL of water, 0.15 g of NaCl, and 50 μL of 1000 ppm methyl cinnamate (internal standard) solution (in ethanol) in 20 mL vials. The equilibration time and temperature were 30 min and 60 °C, respectively. After the equilibration period, the samples were extracted onto 50/30 μm divinylbenzene/carboxen/PDMS for 15 min and then eluted at 220 °C for 10 min. The volatiles were automatically injected into a gas chromatography–mass spectrometry system through a transfer line at 230 °C (6890 N series gas chromatograph-5975 quadrupole mass selective detector; Agilent Technologies, USA). The separation was performed on a 30 m × 0.25 mm i.d. DB-WAX (0.25-μm film) fused silica capillary column (J&W Scientific, USA). The oven program was as follows: 40 °C for 6 min; increase at 4 °C/min to 90 °C; increase at 9 °C/min to 200 °C; and hold for 8 min at 200 °C. The carrier gas (He) velocity was 1.0 mL/min (constant flow). The ionization energy was 70 eV, and the scan range was 50–500 *m/z*. All compounds were identified by comparison with the NIST library (including Wiley and Mainlib) spectral data bank. Only compounds whose similarity was >750 (the maximum similarity is 1000) are reported here. All analyses were performed in triplicate. Quantitative analysis was based on the peak area of a particular component.

### 2.6 Heatmap generation

Heatmaps were drawn to compare and analyze common volatile compounds produced by fungal starters. Using Gitools v2.2.2., heatmaps and hierarchical clusters of the relative proportion and relative abundance of volatile compounds in fermented soybeans were generated [19].

### 2.7 Statistical analysis

Duncan's multiple range test following one-way analysis of variance was used to evaluate significant differences between the average values obtained in volatile compound analyses. Values of *p* < 0.05 were considered statistically significant. To visualize the differences between the volatile compounds produced during soybean fermentation by fungal starters, principal component analysis (PCA) with maximum variation rotation was applied. All statistical analyses were performed using the SPSS software package (version 27.0; IBM SPSS Statistics, USA).

## 3 Results and discussion

### 3.1 Growth of inoculated strains during fermentation

**Fig. 1:**
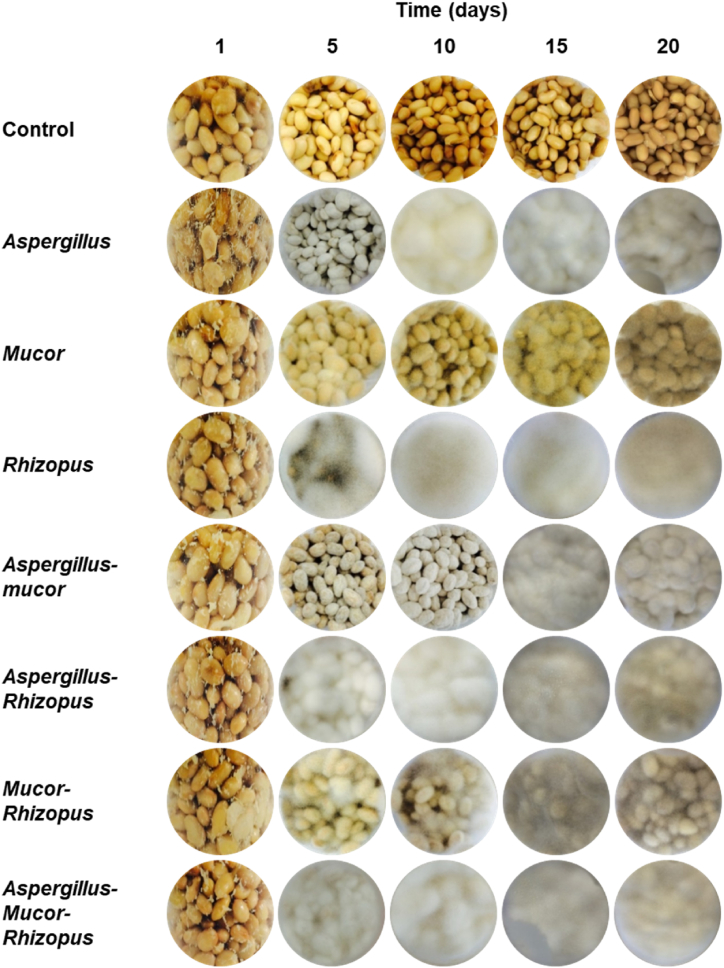
Fungal morphology on soybean fermented with single or mixed strains. Control means soybean without strain.

Test soybean was inoculated with 5.0 × 10^7^ spores/g, which increased slightly during fermentation (Fig. S1); after 20 days, the average spore count in all samples was 1.4 × 10^8^ spores/g. The morphology of fungi in the soybean during fermentation is shown in Fig. 1. The morphologies differed depending on which strains were inoculated. Although soybean added *Mucor*, *Aspergillus*-*Mucor*, or *Mucor*-*Rhizopus*, each of which contained *Mucor* sp. KACC 46077, formed less mycelium than soybeans inoculated with other strains, mycelium increased in all test beans over time. Among soybeans inoculated with a single strain, the most abundant white mycelium was observed in soybean added *Rhizopus*.

### 3.2 Physicochemical constituent analysis of soybean inoculated with single or mixed strains

**Fig. 2:**
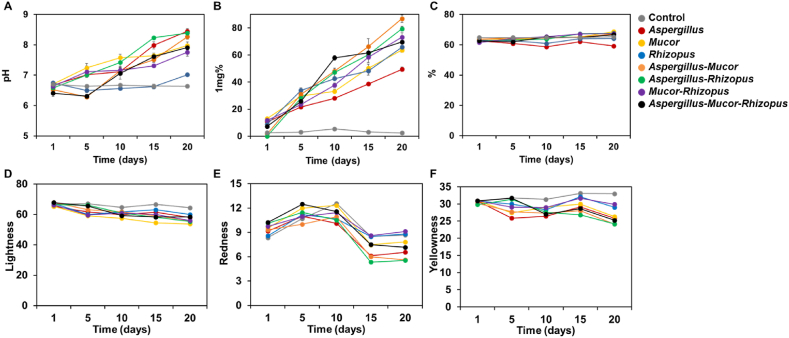
Changes in pH (A), amino-type nitrogen content (B), moisture content (C), lightness (D), redness (E), and yellowness (F) of fermented soybean products.

Production of traditional *doenjang* depends on beneficial microorganisms present in nature. For more scientific industrial production of *doenjang*, the production efficiency must be improved and the fermentation operation made simpler, for example by isolating and inoculating strains with particular, desirable properties. Therefore, in this study, fermented soybean was produced using various inocula (single and mixed strains). Inoculation of soybean with single and mixed strains increased the pH, amino-type nitrogen, and moisture content, and affected the color (Fig. 2).

Changes in pH during the fermentation of the soybeans are shown in Fig. 2A. The pH in inoculated soybean increased by 0.27–1.81 units after a 20-day fermentation period. The pH of soybean added *Aspergillus*-*Rhizopus* increased most (1.81 pH units), followed by soybean added *Aspergillus* (1.78), *Aspergillus*-*Mucor* (1.75), and *Aspergillus*-*Mucors*-*Rhizopus* (1.50). Interestingly, all samples with greatly changed pH contained *A. oryzae*. The pH of soybean added *Rhizopus* increased only slightly. *Aspergillus* and *Mucor* produce protease that is optimally active at neutral or alkaline pH [[20], [21], [22]]. Meanwhile, the protease of *R. oryzae* is optimally active at acidic pH (3–5) [23]. These results suggest that the inoculated strains could be involved in macromolecular degradation.

Fig. 2B shows the changes in the content of amino-type nitrogen. The content of amino-type nitrogen increased as the fermentation period increased, by 28.93–85.09 mg% after a 20-day fermentation. The amino-type nitrogen of soybean added *Aspergillus*-*Mucor* changed most (85.09 mg%), followed by soybean added *Aspergillus*-*Rhizopus* (79.45 mg%), and soybean added *Aspergillus*-*Mucor*-*Rhizopus* (62.29 mg%). Interestingly, these results showed that the mixed strains produced more amino-type nitrogen than the single strains.

Changes in moisture content during the fermentation of soybean by different strains are shown in Fig. 2C. The moisture contents were slightly increased, by 1.3%–6.0%, from 61.5% to 64.8% in the initial stage to 64.2%–68.7% after a 20-day fermentation. Chancharoonpong et al. [24] presumed that the moisture content in *koji* during fermentation was increased because macromolecules in soybean were hydrolyzed by the actions of microorganisms, producing free water. In our study, the moisture contents were slightly increased; the increase in moisture content in soybean added *Aspergillus*-*Mucor* was greatest, which might indicate a synergetic effect between the *A. oryzae* and *Mucor*.

**Fig. 3:**
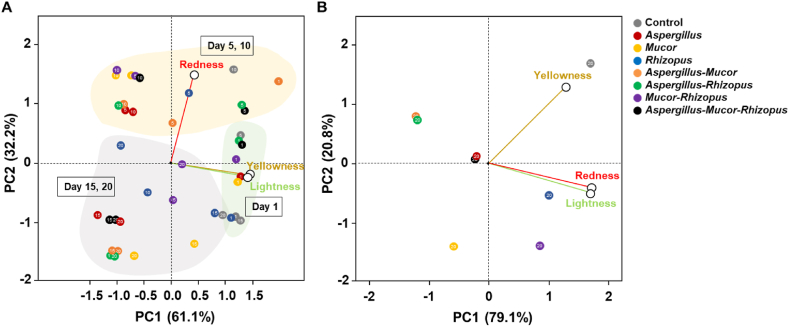
**Principal component analysis (PCA) of color change of fermented soybean during the fermentation period (A) and at day 20 of fermentation (B).** The factors (lightness, redness, and yellowness) used in PCA are indicated by lines in the figure, and the circular spots indicate the score in PCA. Numbers indicate the incubation time of samples in days. (For interpretation of the references to color in this figure legend, the reader is referred to the Web version of this article.)

The color of fermented soybeans is important for consumer acceptance. Changes in color during the fermentation are shown in Fig. 2D–F. The lightness, redness, and yellowness of most samples decreased slightly during fermentation. To identify differences in color changes dependent on the inoculated strains, principal component regression was performed. PCA plots indicated that the color changed according to the inoculated strain(s) and the fermentation time (Fig. 3A). Unusually, the redness increased after 5 and 10 days of fermentation and then decreased. Lightness and yellowness decreased during the fermentation. On the 20th day of fermentation, the color of the fermented soybeans could be distinguished visually according to the inoculated strain(s) (Fig. 3B). Soybean added *Rhizopus* showed less reduction in redness and yellowness compared with other soybean cultures, and soybean added *Mucor* showed a significant reduction in lightness. Soybean added *Aspergillus, Aspergillus*-*Rhizopus, Aspergillus*-*Mucor*, and *Aspergillus*-*Mucors*-*Rhizopus* were all located in the second quadrant of the PCA coordinate place (Fig. 3B). Confirming relationships between the mycelium color of the inoculated strain and the color of the fermented soybean is difficult, but the color of the fermented soybean can be assumed to be affected by the inoculated fungus/fungi. The color of soybean paste has been reported to vary depending on the type of raw material, the fermentation conditions, and the type of fermenting microorganism(s) [25]. The properties of the particular inoculated species and strain should be checked because, for different strains of *A. oryzae*, the color of the spores generated is diverse, and the effect on the color of soybean paste is reported in various method using different strains of *A. oryzae* [26].

### 3.3 Effect of starter cultures on volatile compound production in soybean fermentation

Volatile compounds were analyzed in soybeans fermented for 1 and 20 days to determine the influence of the inoculated strains (Table 1). Forty-two volatile compounds were identified from the fermented soybean, including an acid, alcohols, carbonyls, furans, and others. Compounds present in the control were also present in most fermented samples in different amounts, though some compounds were specific to some inocula. After 20 days of fermentation, compared with the uninoculated soybean as control, the number of volatile compounds was high in the inoculated soybean model: alcohol compounds were abundant in soybean added *Aspergillus*, and *Aspergillus*-*Rhizopus*; and carbonyl compounds were abundant in soybean added *Aspergillus*, *Rhizopus, Aspergillus*-*Rhizopus*, and *Aspergillus*-*Mucor*-*Rhizopus*. Furan compounds were produced at similar concentrations in all the all samples. Generally, volatile compounds (in terms of both number and amount) were abundant in soybean added *Aspergillus* compared with other samples, while soybean added *Mucor* produced the lowest amount of volatile compounds.

**Table 1:** Volatile compounds produced in fermented soybean inoculated with starter candidates. Values presented are the relative peak areas compared with that of the internal standard (average of three replicates). Retention index was calculated based on using a DB-WAX fused silica capillary column.

		Control		*Aspergillus*		*Mucor*		*Rhizopus*		*Aspergillus-Mucor*		*Aspergillus-Rhizopus*		*Mucor-Rhizopus*		*Aspergillus-Mucor-Rhizopus*	
Volatile compound	KI	1 day	20 day	1 day	20 day	1 day	20 day	1 day	20 day	1 day	20 day	1 day	20 day	1 day	20 day	1 day	20 day
**Acid**																	
Ethyl hexadecanoate															0.05^b^	0.14^c^	
**Alcohols**																	
(5*Z*)-Octa-1,5-dien-3-ol	1436				21.33^c^						6.59^b^						
(*E*)-But-2-en-1-ol	1260														0.32^b^		
(*E*)-Hex-3-en-1-ol	1397	0.22^c^	0.08^b^			0.07^b^											
1-Methylbutan-3-ol	1250				0.31^b^												
2-Phenylethanol		0.03^a^		0.02^a^		0.19^ab^	0.08^a^	0.04^a^	0.17^ab^	0.06^a^		0.71^c^		0.23^ab^	0.16^a^	0.39^b^	
3-Hydroxy-2-methylpyran-4-one			0.52^b^														
3-Methylbutan-1-ol	1253	0.54^a^	0.64^a^	2.72^a^		16.02^abc^	10.66^ab^	1.63^a^	9.32^ab^	3.94^ab^	0.29^a^	45.87^d^	31.66^bcd^	19.70^abcd^	31.38^bcd^	24.95^abcd^	40.46^cd^
3-Methylsulfanylpropan-1-ol	1567																0.83^b^
4-Methylpentan-1-ol	1366			0.22^a^								0.45^b^					0.13^a^
5-Methoxypentan-1-ol	1476																0.28^b^
Butan-1-ol	1237														0.15^b^	0.53^c^	
Butan-2-ol	1159		4.83^ab^				3.14^ab^		12.48^c^	3.54^ab^			6.71^b^				
Butane-2,3-diol	1465															0.39^b^	0.56^b^
Hex-3-yn-1-ol	1413																0.31^b^
Hexan-1-ol	1384	7.90^d^	3.49^b^	5.82^c^	0.25^a^	1.37^a^	0.97^a^	1.11^a^	0.78^a^	0.38^a^	0.20^a^	2.32^ab^	1.35^a^	1.31^a^	0.78^a^	0.82^a^	1.37^a^
Oct-1-en-3-ol	1424	11.71^a^	25.02^a^	11.20^a^	224.82^c^	5.11^a^	0.86^a^	7.13^a^		9.01^a^	97.73^b^	12.61^a^	3.41^a^	9.12^a^	0.91^a^	14.56^a^	
Octan-3-ol	1402	0.92^f^	0.28^abcd^	0.52^cd^		0.26^abcd^	0.11^ab^	0.35^bcd^		0.16^ab^		0.58^d^	0.20^abc^				1.37^f^
Pentan-1-ol	1293		1.39^b^				0.23^a^					1.50^b^	0.47^a^		0.64^a^	1.46^b^	
Phenylmethanol																0.05^b^	0.07^c^
**Carbonyls**																	
(2*E*,4*E*)-Nona-2,4-dienal	1265														0.30^b^		
(3*E*)-3-Ethyl-2-methylhexa-1,3-diene	1408								3.84^b^								
(*E*)-Oct-2-enal	1414				1.80^b^				3.37^c^		0.38^a^						
1-(1,3-Thiazol-2-yl)ethanone	1532	0.09^b^						0.18^c^									
1,4-Ditert-butylbenzene	1413		0.12^ab^										0.18^b^				
1-Phenylethanone	1265							1.39^b^									
2-Hydroxy-3-methylcyclopent-2-en-1-one	1519								0.69^b^								
2-Methylbenzaldehyde	1531		0.27^b^														
2-Phenylacetaldehyde	1529	0.11^a^		6.32^c^				0.26^ab^	0.27^ab^	0.85^b^	0.19^ab^	0.11^a^	0.30^ab^		0.13^a^		
Benzaldehyde	1461	0.75^ab^	0.89^b^	0.42^ab^	1.62^c^	0.05^a^	0.06^a^	0.33^ab^	0.31^ab^	0.47^ab^	4.36^d^	0.10^a^	0.65^ab^	0.07^a^	0.28^ab^	0.09^a^	0.52^ab^
Heptan-2-one	1366								0.83^b^								
Heptanal	1217	0.99^b^															
Hexanal	1186	5.92^b^	4.28^ab^	1.62^ab^	2.48^ab^	0.73^a^	0.71^a^	2.97^ab^	13.16^c^	2.25^ab^	3.82^ab^	1.43^ab^	3.45^ab^	0.95^ab^	2.17^ab^	1.28^ab^	2.89^ab^
Nonanal	1399	0.23^b^		0.22^b^			0.17^b^	0.16^b^	0.28^b^								0.22^b^
Oct-1-en-3-one	1419				0.72^a^				2.75^b^								32.75^c^
Octan-3-one	1292	0.80^a^			6.60^c^			0.55^a^	4.64^bc^		3.66^b^						0.16^a^
Octanal	1321								1.56^b^								
Octane-2,3-dione	1368				0.81^b^			0.18^a^	1.35^c^		0.41^ab^						
**Furans**																	
2-Pentylfuran	1270	0.75^bc^	0.89^c^	0.42^abc^	0.54^abc^	0.24^ab^	0.10^a^	0.33^ab^	0.43^abc^	0.36^abc^	0.29^ab^	0.57^abc^	0.55^abc^	0.22^ab^	0.32^ab^	0.38^abc^	0.40^abc^
Furan-2-carbaldehyde	1419			0.42^b^													
Furan-2-ylmethanol	1541	0.11^b^															
**Other**																	
2,3,5-Trimethylpyrazine	1404														0.09^b^		

Different superscripts within a row denote a significant difference between mean values (*p* < 0.05) according to Duncan's multiple range test. Quantitative analysis was based on the peak area of a particular component in gas chromatography-mass spectrometry. KI, Kovats index.

**Fig. 4:**
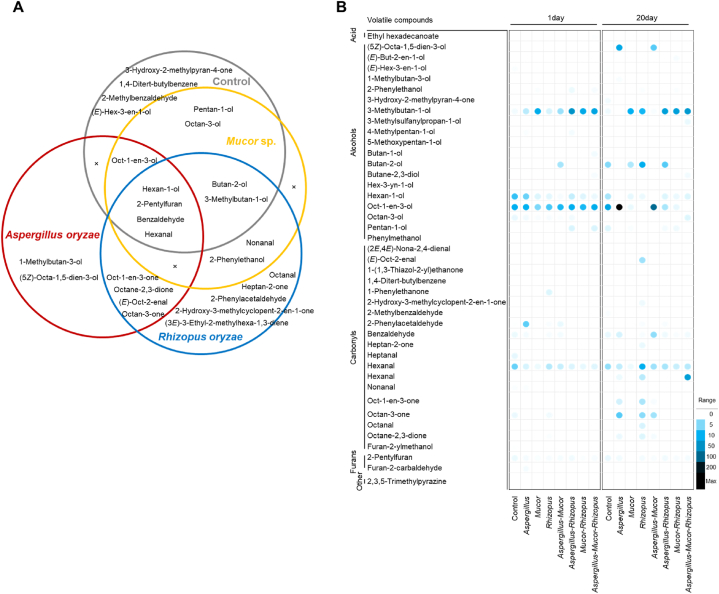
Venn diagram (A) and relative abundance (B) of volatile compounds produced by single fungal strains after a 20-day fermentation of soybean. Overlapping regions of the Venn diagram represent common volatile compounds shared between soybeans fermented with different fungal starters. Each column in the heatmap represents a soybean sample with or without a fungal starter, while each row represents a volatile compound. The color intensity of the panel indicates the relative abundance of the volatile compound. (For interpretation of the references to color in this figure legend, the reader is referred to the Web version of this article.)

We analyzed the distribution of volatile compounds in fermented soybeans inoculated with a single strain and confirmed the contribution of the strains after 20 days of fermentation (Fig. 4). Four compounds—hexan-1-ol, benzaldehyde, hexanal, and 2-pentylfuran—were detected in soybean added *Aspergillus*, *Mucor*, and *Rhizopus*. They were also present, but in lower amounts, in the control; thus, we assumed that those compounds were commonly produced in the fermented soybeans. Oct-1-en-3-ol was detected in the fermented soybeans, except soybean added *Rhizopus*; it was detected in large amounts in soybean added *Aspergillus* as well as in the control. Similarly, butan-2-ol is a volatile compound identified in the control. Starter-specific volatile compounds were detected in this study: 1-metylbutan-3-ol and (5*Z*)-octa-1,5-dien-3-ol in soybean added *Aspergillus*; and 2-hydroxy-3-methylcyclopent-2-en-1-one, 2-phenylacetaldehyde, heptan-2-one, octanal, and (3*E*)-3-ethyl-2-methylhexa-1,3-diene in soybean added *Rhizopus* (Fig. 4A). All volatile components detected in soybean added *Aspergillus*, except (5*Z*)-octa-1,5-dien-3-ol, were previously identified in soybeans fermented by *A. oryzae* [27]. Three of the five volatile compounds specifically detected in soybean added *Rhizopus*—2-phenylacetaldehyde, heptan-2-one, and octanal—were previously in *Aspergillus*-added soybeans [27,28]. However, 2-hydroxy-3-methylcyclopent-2-en-1-one and (3*E*)-3-ethyl-2-methylhexa-1,3-diene have not been found to be produced by *Aspergillus*, and thus we suggest that they are produced specifically by *R. oryzae*.

The 3-methylbutan-1-ol production was increased in mixed culture, especially in soybean added *Aspergillus*-*Mucor-Rhizopus*. During fermentation, 3-methylbutan-1-ol is a commonly identified ingredient and exhibits a fruity odor (Fig. 4B) [27,29]. However, except for this compound, mixed fungi did not significantly increase the type or number of volatile compounds (Table 1).

As mentioned in the Introduction, *Rhizopus* and *Mucor* are fungi that are often found in traditional fermented soybean products in Asia such as *doenjang* in Korea*, miso* in Japan, and *sufu* in China [13,14,[30], [31], [32]]. And there are several results to investigate the correlation between the fungi community of traditional fermented soybeans and the volatile compounds [[31], [32], [33]]. Nevertheless, specific volatile compounds produced by the genus *Rhizopus*, and *Mucor* have not been identified, and there are not as many studies on their potential as there are of *Aspergillus* in starter cultures. Therefore, in this study, the effects on soybean fermentation was compared by inoculation of the three fungi as a single and mixed species. The effect of *Rhizopus* and *Mucor* was difficult to compare with existing studies because of the lack of previous research results. The main result of the present study is that it is possible to infer the effects of these strains on fermented soybeans compared with *Aspergillus*. Fermented soybeans to which *Mucor* sp. KACC 4607 were applied did not produce mycelium abundantly, and did not produce strain-specific volatile components. However, *R. oryzae* KACC 40256 produced abundant mycelium, and two volatile compounds—2-hydroxy-3-methylcyclopent-2-en-1-one and (3*E*)-3-ethyl-2-methylhexa-1,3-diene—were identified when *R. oryzae* was applied. However, further experiments are required to determine whether these compounds were species- or strain-specific. Among them, 2-hydroxy-3-methylcyclopent-2-en-1-one has a fruity-type odor, which is expected to affect the odor of fermented soybeans. Application of *R. oryzae* KACC 40256 also made the product soybeans redder and yellower, which is positive for consumer acceptance. Because starter candidates used for fermentation of soybeans must be able to produce the product desired by the manufacturer, a pool of various starter candidates is required. This study provides basic data toward obtaining desired fermentation products using other species in addition to *A. oryzae*, which will contribute to the production of fermented foods.

## Author contribution statement

Sojeong Heo: Performed the experiments; Analyzed and interpreted the data; Wrote the paper. Junghyun Park: Performed the experiments; Analyzed and interpreted the data. Kwang-Geun Lee: Contributed reagents, materials, analysis tools or data. Jong-Hoon Lee: Conceived and designed the experiments; Contributed reagents, materials, analysis tools or data; Worte the paper. Do-Won Jeong: Conceived and designed the experiments; Analyzed and interpreted the data; Contributed reagents, materials, analysis tools or data; Wrote the paper.

## Funding statement

Do-Won Jeong was supported by 10.13039/501100003725National Research Foundation of South Korea [NRF-2019R1A2C1003639].Jong-Hoon Lee was supported by 10.13039/501100003725National Research Foundation of South Korea [NRF-2022R1F1A1070021].

## Declaration of competing interest

The authors declare that they have no known competing financial interests or personal relationships that could have appeared to influence the work reported in this paper.
