# Supplementary figures and images for: Quality characteristics of soybean fermented by Mucor, Rhizopus, and Aspergillus from meju
Source: Heliyon. 2023 Feb 27;9(3):e14092. doi: 10.1016/j.heliyon.2023.e14092 (PMC10006737; doi:10.1016/j.heliyon.2023.e14092)

**Fig. S1.**


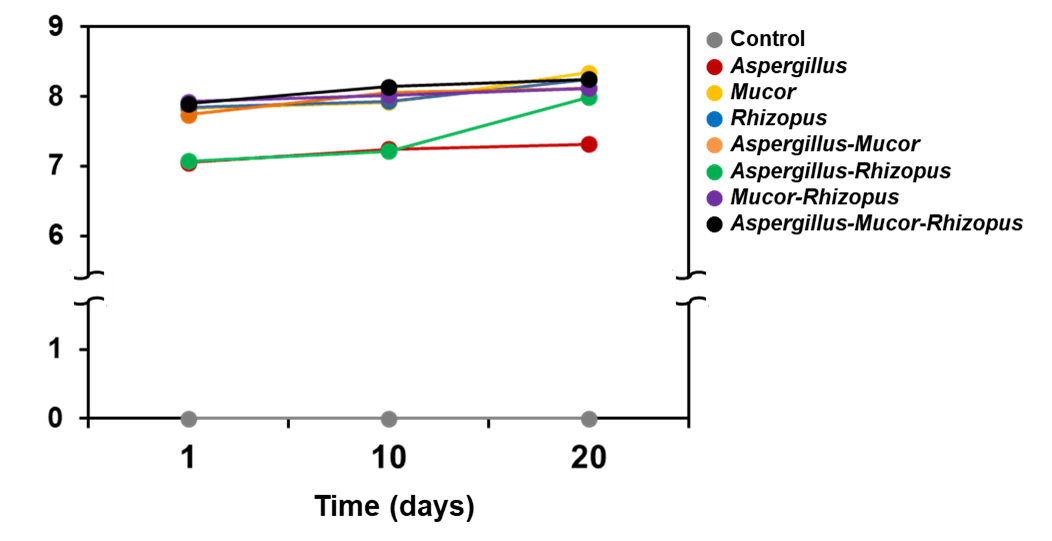

Supplement: Multimedia component 1 [file mmc1.docx]
